# The predictive receiver operating characteristic curve for the joint assessment of the positive and negative predictive values

**DOI:** 10.1098/rsta.2008.0043

**Published:** 2008-04-11

**Authors:** Shang-Ying Shiu, Constantine Gatsonis

**Affiliations:** 1 Institute of Statistical ScienceAcademia Sinica, Taipei 115, Taiwan, Republic of China; 2 Center for Statistical Sciences, Brown UniversityProvidence, RI 02912, USA

**Keywords:** predictive value, PROC curve, ROC curve, diagnostic test

## Abstract

Binary test outcomes typically result from dichotomizing a continuous test variable, observable or latent. The effect of the threshold for test positivity on test sensitivity and specificity has been studied extensively in receiver operating characteristic (ROC) analysis. However, considerably less attention has been given to the study of the effect of the positivity threshold on the predictive value of a test. In this paper we present methods for the joint study of the positive (PPV) and negative predictive values (NPV) of diagnostic tests. We define the predictive receiver operating characteristic (PROC) curve that consists of all possible pairs of PPV and NPV as the threshold for test positivity varies. Unlike the simple trade-off between sensitivity and specificity exhibited in the ROC curve, the PROC curve displays what is often a complex interplay between PPV and NPV as the positivity threshold changes. We study the monotonicity and other geometric properties of the PROC curve and propose summary measures for the predictive performance of tests. We also formulate and discuss regression models for the estimation of the effects of covariates.

## 1. Introduction

Diagnostic tests are evaluated on the basis of measures defined conditionally on the true disease status (sensitivity, specificity and receiver operating characteristic, ROC, curve) or conditionally on the test outcome (positive predictive value, PPV, and negative predictive value, NPV). Measures of test performance, such as sensitivity, specificity or ROC curves, provide the type of information that is typically needed for technology assessment and health policy purposes. Measures of predictive value provide the type of information that is typically needed for clinical decision-making, where clinicians and patients decide whether to use a test or how to assess the implications of a test result. The clinical relevance of predictive value information notwithstanding, a large majority of diagnostic test evaluations continue to be designed with a primary focus on measures defined conditionally on the disease status. One of the reasons for this is the theoretical invariance of sensitivity and specificity to disease prevalence. Predictive values, on the contrary, vary across populations with different disease prevalence, making comparisons of diagnostic tests difficult.

The invariance to disease prevalence of quantities such as sensitivity and specificity is predicated on the assumption that the disease status is the only variable affecting test outcome. Although this simplification is useful as a building block of the theory of diagnostic test evaluation, in reality the test outcome may depend on a number of other characteristics beyond disease status (Moons & Harrel 2003). For example, in a clinical study where electrocardiographic stress test was used to diagnose coronary artery disease, the sensitivity and specificity of this stress test result varied substantially according to gender, relative workload as well as the number of diseased vessels (Moons *et al*. 1997). Moreover, variations in sensitivity, specificity and ROC curves are routinely observed in meta-analysis of diagnostic tests (Irwig *et al*. 1994; Rutter & Gatsonis 2001) and in studies of the performance of test interpreters (Ishwaran & Gatsonis 2000; Beam *et al*. 2003).

The available statistical methodology for the study of the predictive value of tests is less extensive than the corresponding methodology for measures defined conditionally on disease status (Bennett 1985; Pepe 2003). Copas (1999) proposed to use the logit rank plot as a summary of the effectiveness of risk scores. Summary measures of the resulting predictiveness curve were subsequently considered (Bura & Gastwirth 2001) and a thorough study of inference based on the full predictiveness curve was presented (Huang *et al*. 2007). Leisenring *et al*. (2000) discussed a model-based approach to the comparison of the predictive values of binary tests for paired designs. In this approach, a marginal regression modelling framework was used with disease status as the response variable and test indicator as an explanatory variable.

The effect of the threshold value used to declare a positive test result is a fundamental aspect of our understanding of the performance of diagnostic tests. This threshold is the conceptual basis for the well-known trade-off between sensitivity and specificity of a test that gives rise to the ROC curve. A significant body of statistical literature has discussed models with implicit or explicit thresholds for test positivity (Hanley & McNeil 1982; Hanley 1989; Hanley 1998; Pepe 2003). Although it is clear that the PPV and NPV of a test are also functions of the threshold for test positivity, the effect of this dependence has not been studied extensively. This dependence induces a close relation between the two quantities and implies that if the threshold is moved *both* will be affected. It follows that a complete characterization of the predictive power of a test requires the study of both quantities as a pair. Moskowitz & Pepe (2004) proposed a graphical method and a regression framework to estimate and compare predictive values of continuous prognostic factors as a function of the positivity threshold. In that work, the PPV and NPV are quantified and assessed separately. To the best of our knowledge, the *joint* evaluation of the two quantities has not been discussed in the literature. Although both quantities may not be of equal interest in a given practical setting, it is rarely the case that interest lies exclusively in only one of them. However, the joint behaviour of the two quantities cannot necessarily be inferred from their marginal behaviour that would be assessed by a separate analysis of each.

In this paper, we undertake a systematic study of the effect of the positivity threshold on the pair of PPV and NPV of tests. Our emphasis is on the study of the interplay between the two types of predictive values of a test as the positivity criterion varies and on the development of summaries of a test's possible pairs of predictive values. In particular, we define the predictive receiver operating characteristic (PROC) curve that shows all possible pairs of the PPV and NPV of a test as the threshold varies. We study the geometric properties of the PROC curve, discuss methods for estimating the curve for continuous and ordinal valued tests and propose summary measures for the predictive performance of tests. We also formulate and discuss regression models for the estimation of the effects of covariates.

This paper is organized as follows. In §2 we define the PROC curve that can apply to tests with continuous or ordinal categorical outcomes. The geometric patterns and other properties of the PROC curve are discussed in §3. Details of mathematical derivations of PROC curve properties are presented in appendix B of the electronic supplementary material. Section 4 presents the estimation of the PROC curve. We discuss an indirect approach through ROC curve estimation for tests in general. We also propose a direct approach that jointly estimates the PPV and NPV for tests with ordinal outcomes. In §5 we describe methods for evaluating a test's predictive performance and comparing tests using the PROC curve. Illustrative examples with both continuous and ordinal test data are presented in §6, including assessing the ability of standardized uptake value based on lean body mass (SUV-lean; continuous test data) to predict axillary node involvement in women diagnosed with breast cancer and comparing predictive accuracy between digital and screen-film mammography (ordinal test data) for breast-cancer screening. Section 7 summarizes our conclusions.

## 2. Definition: the PROC curve

Let *D* be a binary random variable representing the disease status (0=non-diseased, 1=diseased) with prevalence *p*=*Pr*(*D*=1). Let *T* be a continuous random variable representing the underlying latent scale of test result. We denote by *G* and *F* the cumulative distribution functions of *T*|*D*=1 and 0, respectively. In this notation, the PPV and NPV for a positivity threshold *c* are given by

(2.1) $$\text{PPV}(c)=Pr(D=1|T\geq c)=\frac{[1-G(c)]p}{\left[1-G(c)]p+[1-F(c)](1-p\right)},$$

(2.2) $$\text{NPV}(c)=Pr(D=0|T<c)=\frac{F(c)(1-p)}{G(c)p+F(c)(1-p)}.$$

Analogous to the definition of the ROC curve, the PROC curve is defined as follows:

$${\left\{(1-\text{NPV}(c),\text{PPV}(c))\right\}}_{c\in R},$$

where *R* is the set of all possible thresholds for test positivity. The PROC curve consists of points representing all possible combinations of the PPV and one minus the NPV generated by varying the threshold. This curve displays the interplay between the two quantities and provides a graphical display of the predictive performance of a diagnostic test over the range of possible thresholds. Strictly speaking, the above definition applies to the *theoretical* PROC curve of the test. The *empirical* PROC curve is simply the collection of the observed (1−NPV, PPV) points connected by straight-line segments.

## 3. Properties of predictive values

The ‘ideal’ point in the PROC graph is the point (0, 1) on the *y*-axis, at which both predictive values equal 1. Hence, in analogy to ROC curves, a PROC curve would indicate high predictive performance if small trade-offs in NPV would enable the test to reach high PPV. However, the geometric patterns of PROC curves can often be rather complex, at least in comparison with the patterns observed in ROC curves. It is therefore essential to study and attempt to characterize the geometric properties of PROC curves before undertaking an investigation of how the curves can be used to evaluate the performance of a diagnostic test. This section is devoted to an exploration of properties of the *theoretical* PROC curve, with a detailed investigation of the commonly used binormal ROC model, which assumes that some monotone increasing transformation of the test result follows a standard normal distribution, conditional on *D*=0, and follows a normal distribution with mean *a* and standard deviation *b*, conditional on *D*=1. Equivalently, parameter *a* represents the difference between the two means and parameter *b* represents the ratio of two standard deviations of the underlying normal distributions.

### (a) PROC curve as a function of disease prevalence

In contrast to the ROC curve that is invariant to disease prevalence, the geometric characteristics of the PROC curve are partially determined by disease prevalence. As can be seen from (2.1) and (2.2), the positive predictive value increases when the prevalence *p* increases and the negative predictive value decreases. In other words, with an increasing prevalence, a point on a PROC curve moves towards its upper-right direction.

**Figure 1:**
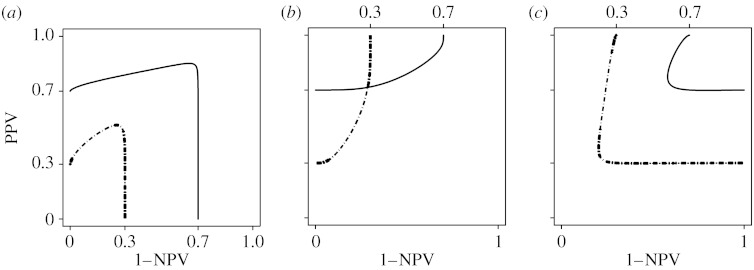
Predictive curves with *a*=0.8. (*a*) *b*=0.7, (*b*) *b*=1, (*c*) *b*=1.5. Solid line, high prevalence (*p*=0.7); dot-dashed line, low prevalence (*p*=0.3).

The two extreme points (when the positivity threshold approaches minus infinity and infinity) of a curve correspond to settings in which all cases are classified as either positive or negative. Unlike the ROC curve in which the extreme points are always (0, 0) and (1, 1), the PROC curve has extreme points that also depend on the prevalence. Figure 1 shows examples under the usual binormal ROC model for selected values of *a*, *b* and *p*. Assuming *a* and *b* fixed, the prevalence affects primarily the location of the curve and only secondarily the shape of the curve.

### (b) Monotonicity of the PROC curve

Perhaps the most challenging complexity of PROC curves arises from the potential that two distinct values of PPV can correspond to the same value of NPV and conversely. To examine this issue, we begin by defining a *monotonic* PROC curve as a curve that has a one-to-one correspondence between the PPV and NPV. Lack of monotonicity is extensively present even in the binormal ROC model. Monotonicity can hold globally, over all threshold values, or locally, over particular intervals of the threshold values. If the PROC is seen as the graph of PPV as a function of NPV, our definition of monotonicity corresponds to the usual notion of monotonicity of a function. Before discussing the definition of summary measures and optimal operation points, the issue of the monotonicity of the PROC curve needs to be addressed.

A mathematical characterization of monotonicity can be obtained by considering properties of the hazard rate. By taking the first derivatives of predictive values in (2.1) and (2.2) as given in §2, we obtain the following two inequalities:

(3.1) $$\frac{\text{d}}{\text{d}c}\;\text{PPV}(c)\geq 0\Leftrightarrow \frac{g(c)}{1-G(c)}\leq \frac{f(c)}{1-F(c)}$$

and

(3.2) $$\frac{\text{d}}{\text{d}c}\;\text{NPV}(c)\geq 0\Leftrightarrow \frac{g(c)}{G(c)}\leq \frac{f(c)}{F(c)}.$$

Pairs of random variables with cumulative distribution functions and densities satisfying inequality (3.1) for all *c* are said to be *hazard rate ordered*, while pairs satisfying (3.2) for all *c* are said to be *reversed hazard rate ordered* (Shaked & Shanthikumar 1994). A random variable *X* is smaller than *Y* in the hazard rate order if the slope of the logarithm of the survival function of *X* is uniformly smaller than the corresponding slope for *Y*. Further discussion and an example of when the condition does not hold are provided in appendix A of the electronic supplementary material. Returning now to the discussion of the monotonicity of the PROC curve, a necessary and sufficient condition for the PPV and NPV to be monotone is that *T*|*D*=1 and 0 are hazard rate ordered and reversed hazard rate ordered, respectively. Both hazard rate order and reversed hazard rate order are guaranteed by the *likelihood ratio order*, under which the ratio *f*(*c*)/*g*(*c*) is a monotone function of *c*. The above implies that a sufficient condition for the monotonicity of the PROC curve is that the pair of, possibly latent, test result variables for truly positive and negative cases is likelihood ratio ordered. This property of monotone likelihood ratio is possessed by the well-known one-parameter exponential family with a monotone canonical link function. Examples include exponential distributions, gamma distributions with the same shape parameters and logistic distributions with the same scale parameters.

We now investigate the monotonicity property for the commonly used binormal model. Technical details of the derivation are provided in appendix B of the electronic supplementary material. Proposition 3.1 identifies values of the parameters for which the binormal PROC curve is monotone.

Proposition 3.1 *Assume that a*>0. *For b*=1*,*

- the positive predictive value increases as the threshold increases and
- *the negative predictive value decreases as the threshold increases*.

Proposition 3.1 shows that the PROC curve is not only monotone, but also it shows a trade-off between PPV and NPV if and only if *b*=1. When *b*≠1, we can consider only intervals of positivity threshold over which the PROC curve segments are monotone.

Proposition 3.2 *Assume that a*>0.

- *For b*>1*,*
  - *the positive predictive value is strictly decreasing on* $c\in (-\infty ,c_{\text{PPV}}^{*})$ *and is strictly increasing on* $c\in (c_{\text{PPV}}^{*},\infty )$ *and*
  - *the negative predictive value is strictly increasing on* $c\in (-\infty ,c_{\text{NPV}}^{*})$ *and is strictly decreasing on* $c\in (c_{\text{NPV}}^{*},\infty )$.
- *For b*<1*,*
  - *the positive predictive value is strictly increasing on* $c\in (-\infty ,c_{\text{PPV}}^{*})$ *and is strictly decreasing on* $c\in (c_{\text{PPV}}^{*},\infty )$ *and*
  - *the negative predictive value is strictly decreasing on* $c\in (-\infty ,c_{\text{NPV}}^{*})$ *and is strictly increasing on* $c\in (c_{\text{NPV}}^{*},\infty )$*,*

*where* $c_{\text{PPV}}^{*}$ *and* $c_{\text{NPV}}^{*}$ *are unique solutions of*

$$\frac{\frac{\phi \left(\frac{c-a}{b}\right)}{b}}{1-\Phi \left(\frac{c-a}{b}\right)}=\frac{\phi (c)}{1-\Phi (c)}$$

*and*

$$\frac{\frac{\phi \left(\frac{c-a}{b}\right)}{b}}{\Phi \left(\frac{c-a}{b}\right)}=\frac{\phi (c)}{\Phi (c)},$$

*respectively. Moreover,* $c_{\text{PPV}}^{*}\leq c_{\text{NPV}}^{*}$.

Proposition 3.2 shows that both the PPV and NPV have only one local maximum (or minimum). We denote these positivity thresholds as $c_{\text{PPV}}^{*}$ and $c_{\text{NPV}}^{*}$. The PROC curve is monotone and shows a trade-off between the PPV and NPV on $c\in (-\infty ,c_{\text{PPV}}^{*})$ and $c\in (c_{\text{NPV}}^{*},\infty )$. On $c\in (c_{\text{PPV}}^{*},c_{\text{NPV}}^{*})$, the curve is also monotone but does not show such a trade-off. Indeed both the PPV and NPV either decrease (when *b*<1) or increase (when *b*>1) as the positivity threshold traverses this interval.

**Figure 2:**
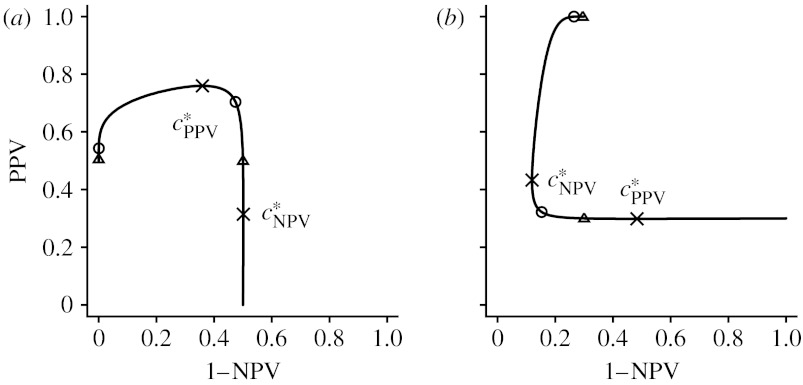
Each non-monotone PROC curve consists of three monotone curve segments defined on $\left(-\infty ,c_{\text{PPV}}^{*}\right)$, $\left[c_{\text{PPV}}^{*},c_{\text{NPV}}^{*}\right]$ and $\left(c_{\text{NPV}}^{*},\infty \right)$, respectively. Circles denote points corresponding to operating thresholds at −1 and *a*+*b*, triangles denote points corresponding to operating thresholds at −2 and *a*+2*b* and crosses denote $c_{\text{PPV}}^{*}$ and $c_{\text{NPV}}^{*}$. (*a*) *a*=1, *b*=0.5, *p*=0.5; (*b*) *a*=2, *b*=2, *p*=0.3.

Figure 2 gives examples of PROC curves for selected values of *a* and *b*≠1. We note that some parts of the PROC curve, even though identified as strictly increasing or decreasing by proposition 3.2, are visually vertical or horizontal lines. The presence of these line segments is due to the fact that one of the PPV or NPV converges much faster than the other as the threshold approaches the infinity or the minus infinity. Moving the positivity threshold along these lines does not influence one of the PPV or NPV, while the other predictive value decreases (or increases). Figure 2 also locates the points at which the operating thresholds are ±1 and ±2 s.d. (of the distributions for the diseased and the non-diseased) away from the means. Note that a considerable part of the PROC curve corresponds to threshold values beyond ±2 s.d.

To summarize, propositions 3.1 and 3.2 give monotonicity properties of the PROC curve. For tests with either continuous or ordinal outcome, the parameters of this curve can be estimated and the monotonicity of the estimated curve can be assessed. In practice, since a considerable part of the PROC curve may correspond to threshold values outside the usual range of interest, a partial PROC curve is often of more interest than a whole curve. This curve segment of interest may well be monotone.

### (c) Further comments on the interpretation of the PROC curve

**Figure 3:**
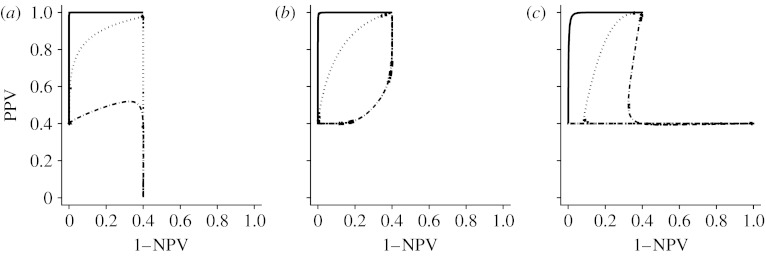
The movement of the PROC curves with increasing *a*'s. (*a*) *b*=0.7, (*b*) *b*=1 and (*c*) *b*=1.5. Solid line, *a*=5; dotted line, *a*=2; dot-dashed line, *a*=0.5.

We note that the shape and location of the PROC curve are naturally affected by the degree of separation in the distribution of test results between positive and negative cases. Figure 3 shows the effect of this separation in the binormal model.

**Figure 4:**
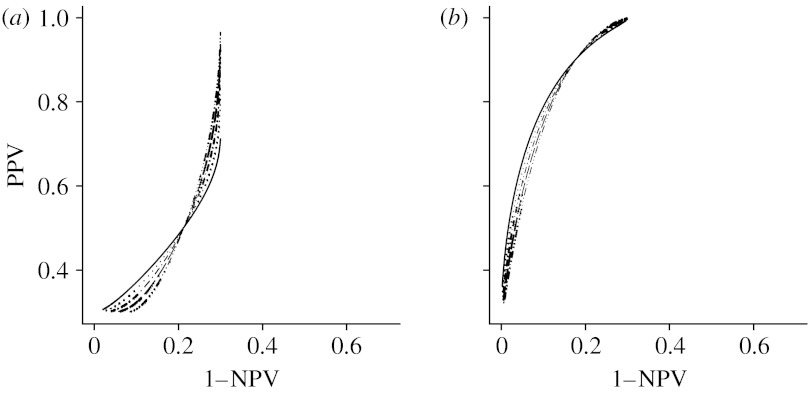
Each PROC curve segment corresponds to c∈[−3,a+3b], where the prevalence is 0.3. (*a*) *a*=0.8, (*b*) *a*=2. Solid line, *b*=0.9; dotted line, *b*=0.95; dot-dashed line, *b*=1; dashed line, *b*=1.05; triple dot-dashed line, *b*=1.1.

From propositions 3.1 and 3.2, we also note that the patterns of the shapes of the PROC curves can be summarized by three categories, which are patterns of *b*>1, *b*=1 and *b*<1, as shown in figure 1. The apparent discontinuity of the binormal curve at the point *b*=1, however, is the result of the behaviour of the curve at the extremes of the range of possible thresholds. Figure 4 shows PROC curve segments corresponding to threshold values on [−3, *a*+3*b*] from *b*<1 to *b*>1 for selected values of *a*. In contrast to the whole curve, the curve segments in the figure show a continuous pattern of change.

## 4. PROC curve estimation

A convenient way to derive estimates of the PROC curve is by first estimating the ROC curve and the prevalence for a set of data and then using a suitable transformation. The joint likelihood of the test outcome and the true disease status can be factored into two terms

$$L_{\left(T,D\right)}=L_{T|D}L_{D},$$

where *L*_*T*|*D*_ is constructed from the distribution of the test outcome conditional on the disease status and *L*_*D*_ is from the marginal distribution of the disease status. *L*_*T*|*D*_ and *L*_*D*_ have mutually exclusive parameter sets; therefore, the maximization of *L* is equivalent to maximize *L*_*T*|*D*_ and *L*_*D*_ separately. The conditional distribution of test results expressed by *L*_*T*|*D*_ has been used for deriving the estimates of sensitivity, specificity and ROC curve (Tosteson & Begg 1988; Toledano & Gatsonis 1996; Metz *et al*. 1998; Pepe 1998, 2000*b*). The distribution of true disease status, *L*_*D*_, is of interest in prospective studies and can also be modelled as a function of covariates.

In this section we propose an alternative approach for PROC curve estimation when the diagnostic test is ordinal categorical. Instead of an indirect approach through the ROC curve estimation, we consider direct modelling of the two types of predictive values through a joint formulation. The model is henceforth referred to as *the predictive model*. Parameter estimation is based on a pseudo-likelihood approach. Estimates of the PROC curve can be obtained by parameter estimates from the predictive model. Models for regression analysis of PPV and NPV separately have been proposed in the literature (Leisenring *et al*. 2000; Moskowitz & Pepe 2004; Huang *et al*. 2007). The formulation here treats the two quantities jointly.

### (a) The predictive model: model specification

Let T^*^ be a random variable representing the test result taking ordered categories 1, 2, …, *K* (1=definitely normal, *K*=definitely abnormal). Then $\left\{T^{*}\geq k\}=\{T\geq \theta_{k}\right\}$, where *θ*_*k*_'s denote the positivity thresholds on the underlying continuous scale *T*. Combining (2.1) and (2.2), under the usual binormal assumption, we obtain

$$Pr(D=1|I_{k})=\frac{1}{1+rg(\theta_{k},I_{k},a,b)},$$

where $I_{k}=I_{\left\{T^{*}\geq k\right\}}$ is the binary test result based on the *k*th positivity threshold, *r*=(1−*p*)/*p* and $g(\theta_{k},I_{k},a,b)=(I_{k}-\Phi (\theta_{k}))/(I_{k}-\Phi ((\theta_{k}-a)/b))$. This predictive model jointly specifies the PPV and NPV. When *I*_*k*_=1 and 0, the PPV and NPV are modelled, respectively.

The predictive model can be extended to include covariates such as those representing subject characteristics that may influence the predictive performance of a diagnostic test. In studies with more than one test, covariates can also be test indicators, thus making it possible to use the predictive model for test comparisons. Since the predictive performance of a diagnostic test depends on the location parameter *a*, the scale parameter *b* and the prevalence *p*, covariates may affect any one of these three parameters. The predictive model with covariates $x=(1,x_{1},\ldots ,x_{J})$ is defined as

(4.1) $$Pr(D=1|I_{k},x)=\frac{1}{1+(\mathrm{rx})g(\theta_{k},I_{k},\mathrm{ax},\mathrm{bx})}.$$

In the following, we will present a pseudo-likelihood approach for estimating parameters in the predictive model assuming independent observations.

### (b) Formulation of the pseudo-likelihood function

Let *I*_*k*_ be as defined in §4*a*, and let PPV_*k*_ and NPV_*k*_ be the PPV and NPV, respectively, using the *k*th positivity threshold. The disease status conditional on the test result follows a Bernoulli distribution

$$D|I_{k}∼\text{Bernoulli}(q_{k})\forall k,$$

with $q_{k}={\text{PPV}}_{k}I_{k}+(1-{\text{NPV}}_{k})(1-I_{k})$. Assuming that each individual *i* is independent, the log-likelihood function for each positivity threshold *k* is

$$ll_{k}(a,b,r,\theta_{k})=\sum_{i=1}^{N}\text{ln}\;f_{k}\;(D_{i}|I_{ik})=\sum_{i=1}^{N}D_{i}\;\text{ln}\;(q_{ik})+(1-D_{i})\;\text{ln}\;(1-q_{ik}).$$

According to the predictive model (4.1), *ll*_*k*_ can be written as

$$ll_{k}(a,b,r,\theta_{k})=\sum_{i=1}^{N}-D_{i}\;\text{ln}\;(1+h_{ik})+(1-D_{i})\;\text{ln}\;\left(1-\frac{1}{1+h_{ik}}\right),$$

where $h_{ik}=rx_{i}g(\theta_{k},I_{ik},ax_{i},bx_{i})$.

For each *ll*_*k*_, we can use the maximum-likelihood method to obtain estimates of parameters ***a***, ***b***, ***r*** and *θ*_*k*_. However, since parameters ***a***, ***b*** and ***r*** appear in all *ll*_*k*_'s, maximum-likelihood solutions based on each *ll*_*k*_ are likely to differ. To address this problem, we formulate a single likelihood function consisting of all *ll*_*k*_'s, and therefore will lead to a single set of parameter estimates. This likelihood function can be constructed by the following data replication.

Let each individual be replicated *K*−1 times as if there were another *K*−1 identical individuals in the data, except that the result of test positivity at each time of the replication is based on one of another *K*−1 positivity thresholds. Denote by $R_{i}=(R_{i1},\ldots ,R_{iK})$ the disease status of individual *i* after replication, where $R_{ik}=D_{i}$ for all *k*'s. Denote by $I_{i}=(I_{i1},\ldots ,I_{ik})$ the results of test positivity of individual *i* after replication. Then the log-likelihood function of the replicated data is

(4.2) $$\sum_{i=1}^{N}\text{ln}\;f(R_{i1},\ldots ,R_{iK}|I_{i1},\ldots ,I_{iK}),$$

where $f(R_{i1},\ldots ,R_{iK}|I_{i1},\ldots ,I_{iK})$ is the joint density of *R*_*i*_ given the results of test positivity *I*_*i*_. Since by replication we introduce within-subject correlations, the joint density of *R*_*i*_|*I*_*i*_ is difficult to obtain. Hence, we consider the following approach to bypass the specification of the full likelihood.

Referring to the definition of the log of the pseudo-likelihood function (Aerts *et al*. 2002), we construct the following one for (4.2):

(4.3) $$\sum_{i=1}^{N}\sum_{k=1}^{K}\delta_{k}\;\text{ln}\;f_{k}(D_{i}|I_{ik})=\sum_{k=1}^{K}\delta_{k}ll_{k}.$$

In this formulation, setting δ_*k*_=1 ∀*k* gives equal weight to each positivity threshold. Different weights may be desirable in some study settings.

### (c) Maximum pseudo-likelihood estimators

The maximum pseudo-likelihood estimators are defined as the maximizer of (4.3). Under standard regularity conditions on *f*_*k*_(*D*|*I*_*k*_), the estimators are consistent and asymptotically normal with empirically corrected variance *J*^−1^*QJ*^−1^ (Arnold & Strauss 1991; Aerts *et al*. 2002), where the matrix *J* is the negative matrix of expected second derivatives of the log pseudo-likelihood function, and the matrix *Q* is the expected product of the pseudo-likelihood scores. The standard regularity conditions are to ensure the existence of maximum pseudo-likelihood estimates and a positive definite matrix *J*, which are identified and proved in appendix C of the electronic supplementary material.

Note that although in §4*b* we presented the pseudo-likelihood approach as assuming independent observations, the same approach can easily apply to multiple correlated observations, such as data from studies of paired design. The empirically corrected variance can handle correlations introduced by data replication, as well as the within-subject correlations.

### (d) Estimating predictive values and the PROC curve

Using the maximum pseudo-likelihood estimators with corrected standard errors as described in §4*c*, we can estimate the covariate-specific PPV and NPV by

$$\hat{{\text{PPV}(x)}_{k}}=\frac{1}{1+\hat{r}x\frac{1-\Phi ({\hat{\theta }}_{k})}{1-\Phi \left(\frac{{\hat{\theta }}_{k}-\hat{a}x}{\hat{b}x}\right)}},\;\hat{{\text{NPV}(x)}_{k}}=1-\frac{1}{1+\hat{r}x\frac{\Phi ({\hat{\theta }}_{k})}{\Phi \left(\frac{{\hat{\theta }}_{k}-\hat{a}x}{\hat{b}x}\right)}}.$$

Similarly, we can estimate the covariate-specific PROC curve by

$${\left\{\left(\frac{1}{1+\hat{r}x\frac{\Phi (c)}{\Phi \left(\frac{c-\hat{a}x}{\hat{b}x}\right)}},\;\frac{1}{1+\hat{r}x\frac{1-\Phi (c)}{1-\Phi \left(\frac{c-\hat{a}x}{\hat{b}x}\right)}}\right)\right\}}_{c\in R}.$$

The standard errors of$\hat{{\text{PPV}(x)}_{k}}$,$\hat{{\text{NPV}(x)}_{k}}$ and the estimated PROC curve can be calculated using either the delta method, or bootstrap methods such as sampling with replacement from the original data and parametric bootstrap when the sample size is small.

## 5. The analysis of PROC curves

This section presents methods in evaluating and comparing the predictive performance of diagnostic tests by using PROC curves.

When a PROC curve or a segment of interest is monotone, summary measures such as the area under the full curve (full area) or a particular segment (partial area) can be used to summarize the predictive performance of a diagnostic test. The (partial) area can be interpreted as the average positive predictive value corresponding to a given range of negative predictive value. Comparisons of area under the curves can be made by the delta method.

In addition to summary measures that integrate over ranges of threshold values, we discuss approaches for defining ‘optimal’ points on the PROC curve and corresponding summary indices. A potential advantage of these approaches is that they may be interpretable in both monotone and non-monotone curve settings. As in the case of ROC analysis, it is possible to estimate test values that correspond to the optimal points on the curve using either the method for estimating the curve, for example as in Metz *et al*. (1998), Pepe (2000*a*) and Alonzo & Pepe (2002), or regression models as described in Zhou *et al*. (2002).

### (a) The r^*^ global measure of predictive performance

We begin by proposing a statistic that measures how far away a diagnostic test is from perfect prediction, i.e. when both the PPV and NPV are equal to one. For each threshold *c* and the corresponding point on the PROC curve, this statistic calculates a distance from the point to the ideal point at the left upper corner where (1−NPV,PPV)=(0,1). Explicitly,

(5.1) r(c)=[1−PPV(c)]+[1−NPV(c)].

We also define a statistic r^*^ as the minimum distance between the PROC curve and the point of perfect prediction,

$$r^{*}=\underset{c}{\text{min}}r(c).$$

The statistic r^*^ can be interpreted as the best performance that a diagnostic test can achieve and

$$c^{*}=\text{arg}\;\underset{c}{\text{min}}r(c)$$

is the optimal operating threshold. Note that both r^*^ and c^*^ depend on the prevalence.

An alternative derivation of r^*^ can be obtained as follows. Rewrite (5.1) as

(5.2) PPV(c)=[1−NPV(c)]+1−r(c).

Equation (5.2) represents a straight line on the PROC space with intercept equal to one minus *r*(*c*) and slope equal to one. By minimizing *r*(*c*), we maximize the intercept of this straight line. In other words, r^*^ can be obtained by moving this 45° line towards the upper left corner, until it reaches the highest point that intersects with the PROC curve.

The definition of *r*(*c*) implicitly assumes that a gain in positive predictive value can make up for an equal amount of loss in negative predictive value and vice versa. However, there are situations when either the positive or negative predictive value is more important than the other. To accommodate such situations, the definition of *r*(*c*) can be generalized to

(5.3) $$r_{\alpha }(c)=[1-\text{PPV}(c)]+\alpha [1-\text{NPV}(c)],$$

where *α* indicates the ratio of the penalty due to the loss in PPV and NPV.

Figure 7 shows the distance to perfect prediction as a function of positivity threshold from the SUV-lean data that will be presented in §6*a*. Such a graph can provide valuable descriptive information about the behaviour of *r* in a particular range of threshold values and the overall variation of predictive performance. If in practice it is impossible to set a threshold explicitly, as is the case in reader interpretations of diagnostic imaging, a test with smaller variation in predictive performance might be preferable.

Figure 7 also presents information about r^*^, the best predictive performance of a diagnostic test, as well as the optimal operating threshold on which the best performance is achieved. In practice, we need to evaluate whether the optimal threshold value is realistic. We also need to evaluate how far the test performance, under design settings that are currently the most common, is from the test's optimal performance. Moreover, it is useful to know how much moving the threshold influences the predictive performance. If predictive performance does not increase much by the change of the positivity threshold, then we should evaluate whether clinically it is worth the effort to choose and operate at the optimal threshold to gain only a certain increase in predictive accuracy.

### (b) Average of predictive performance

A summary measure of predictive performance over a range of positivity threshold is provided by the integrated value of *r*(*c*)

(5.4) $$R_{\alpha }=\int r_{\alpha }(c)f(c)\;\text{d}c,$$

where *f* is a weight function defined over values of the positivity threshold. If all positivity thresholds were equally important over a chosen interval, a uniform distribution over the interval would be appropriate. For a continuous diagnostic test, the weight function can be chosen as the empirical distribution of observed test results. For that choice, the average performance *R* is identical to two minus the sum of the areas under the PPV curve and the NPV curve proposed by Moskowitz & Pepe (2004). Note that 0≤*r*_*α*_(*c*)≤1, and *R*_*α*_ is therefore bounded by 1+*α*. *R*_*α*_ represents the average loss from perfect prediction, with smaller values indicating better average predictive performance.

### (c) Comparison between tests

Differences in the predictive performance of tests may be due to differences in disease prevalence under which the tests were evaluated. This type of confounding is addressed automatically in studies using a paired design. In order to compare predictive performance estimates from other types of studies, an adjustment for differences in prevalence is needed. A PROC curve obtained in one population can be transformed to apply to a population with a different prevalence using the following formulae:

$$y_{2}(c)=\frac{y_{1}(c)(1-p_{1})p_{2}}{y_{1}(c)(1-p_{1})p_{2}+(1-y_{1}(c))p_{1}(1-p_{2})},$$

$$x_{2}(c)=\frac{x_{1}(c)(1-p_{1})p_{2}}{x_{1}(c)(1-p_{1})p_{2}+(1-x_{1}(c))p_{1}(1-p_{2})},$$

where $\Gamma_{1}(c)=\{(x_{1}(c),y_{1}(c))\}$ is the PROC curve of some test performed on a population with disease prevalence *p*_1_, and $\Gamma_{2}(c)=\{(x_{2}(c),y_{2}(c))\}$ is the PROC curve of the same test performed on a population with disease prevalence *p*_2_. This transformation is useful in comparisons of diagnostic tests applied to different populations.

We can compare tests on the basis of *r*_*α*_(*c*), as well as on the basis of the average predictive performance *R*_*α*_. Denote by $r_{\alpha }^{\text{A}}(c)$ and $r_{\alpha }^{\text{B}}(c)$ the *r*_*α*_(*c*) statistic of test A and test B, respectively. We can observe whether the confidence bands for *r*^A^(*c*) and *r*^B^(*c*) overlap over a threshold range of particular interest. Similarly, we can observe whether the CIs for $r_{\alpha }^{\text{A}*}$ and $r_{\alpha }^{\text{B}*}$, or $R_{\alpha }^{\text{A}}$ and $R_{\alpha }^{\text{B}}$, overlap. If one test is uniformly better than the other over a specific range of threshold, or at a threshold value of particular interest, then we should further evaluate whether these thresholds are reasonable for conducting clinical studies.

## 6. Examples

To illustrate the ideas and methods developed in this paper, we present the following two examples. The first example used data from a prospective multi-centre study (Walh *et al*. 2004) to evaluate the predictive accuracy of SUV-lean in detecting axillary nodal metastases in women with primary breast cancer. The second example used data from the Digital Mammographic Imaging Screening Trial (DMIST; Pisano *et al*. 2005) to assess and compare the predictive performance of digital and film mammography for breast cancer detection. In both studies, participants were recruited prospectively and the estimates of the PPV and NPV generalize directly to the target populations.

### (a) PROC analysis for SUV-lean data

**Figure 5:**
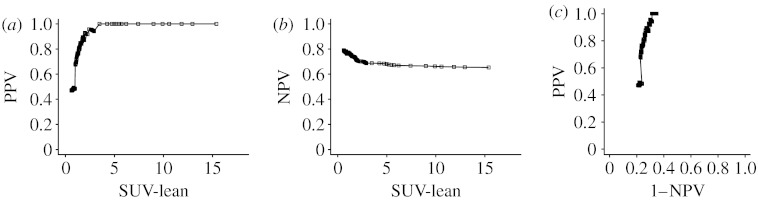
The empirical predictive performance of SUV-lean. (*a*) Positive predictive value; (*b*) negative predictive value; (*c*) PROC curve.

In this study, 360 women with newly diagnosed invasive breast cancer were enrolled and imaged with FDG-PET. We used a subset of 308 cases with assessable axillae for this analysis. SUV-lean, which is a quantitative measurement of the single-pixel maximal standardized uptake value normalized to lean body mass, was determined only for patients with PET imaging score at least 2 (2=equivocal; 3=probably abnormal; 4=definitely abnormal). For patients with PET imaging score 0 (=normal) or 1 (=probably normal), the SUV-lean was imputed by 0.6 and 1. The choice of imputed values was made by the investigators based on the past experience with normal or probably normal cases. The reference standard information for the actual presence of axillary node involvement was derived from surgical exploration of the axilla and subsequent patient follow-up. Figure 5 plots the empirical predictive accuracy of the SUV-lean in terms of the PPV, NPV and the PROC curve.

**Figure 6:**
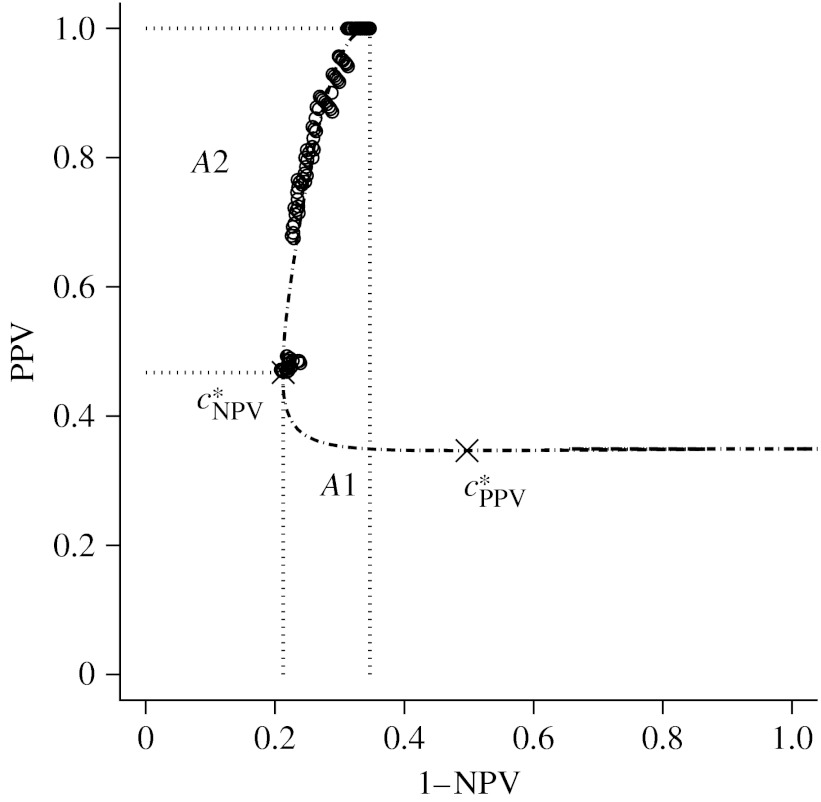
The estimated PROC curve of SUV-lean (dot-dashed line). The circles indicate the pairs of empirical PPV and NPV.

To estimate the PROC curve, we first estimated the ROC curve of the continuously distributed SUV-lean using the maximum-likelihood method proposed by Metz *et al*. (1998). With parameter estimates $\hat{a}=1.134$ (s.e.=0.205) and $\hat{b}=1.704$ (s.e.=0.261), figure 6 presents the estimated PROC curve. The curve was derived from the likelihood method on the joint distribution of *T* and *D*, as described in the beginning of §4.

We are interested in the curve segment that corresponds to the range of observed SUV-lean. This segment has a range of positive predictive value [0.468,1] and a range of negative predictive value [0.653,0.791]. According to proposition 3.2 in §3*b*, this curve segment is monotone and displays trade-off between the PPV and NPV. Therefore, for this single dataset, we can use summary measures analogous to those developed for ROC analysis. The areas under this curve segment are *A*1=0.113 that corresponds to an average positive predictive value of 0.819 and *A*2=0.116 that corresponds to an average negative predictive value of 0.782. These results suggest that the positive and negative predictions by SUV simultaneously reach a reasonably satisfactory level of accuracy.

**Figure 7:**
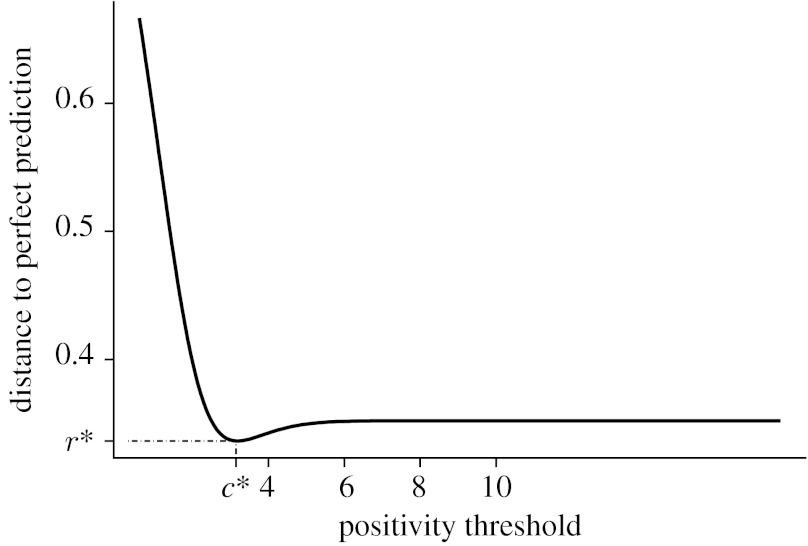
The estimated distance function of SUV-lean.

In addition to the average predictive performance, we also determine the optimal operating point at which the SUV-lean achieves its best predictive performance. Figure 7 shows the performance of SUV-lean by the cut-off point. The optimal performance occurred at 3.23 on the latent scale, which corresponds to an SUV-lean cut-off of 5.13. At the optimal point, the positive predictive value was 0.990 with 95% CI (0.975, 0.996) and the negative predictive value was 0.674 with 95% CI (0.614, 0.728). The small variation of *r*(*c*) on the neighbourhood of c^*^ suggests that the performance of SUV-lean is insensitive to the estimation errors in this optimal threshold.

### (b) Application to DMIST data

**Figure 8:**
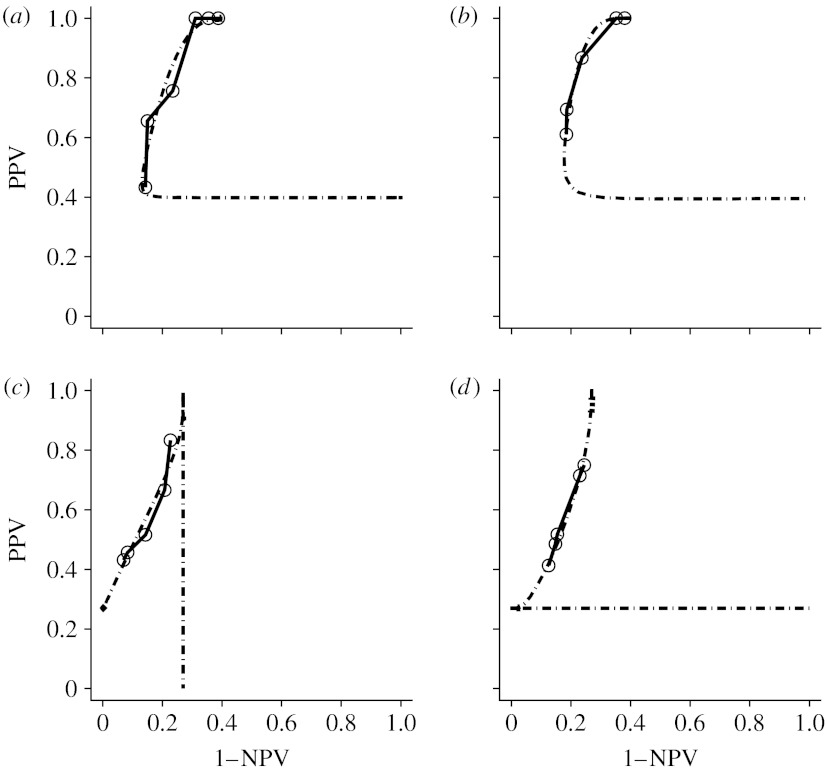
The empirical (solid line) and estimated (dot-dashed line) PROC curves of digital and screen-film mammography from three readers. The circles represent the pairs of empirical PPV and NPV. The number of participants, the prevalence and the estimates of parameters *a* and *b* are also provided. (*a*) Reader 1 film (*N*=118, *p*=0.398, *a*=1.749, *b*=1.537); (*b*) reader 2 film (*N*=119, *p*=0.395, *a*=1.882, *b*=1.927); (*c*) reader 3 film (*N*=94, *p*=0.269, *a*=1.231, *b*=0.918); (*d*) reader 3 digital (*N*=94, *p*=0.269, *a*=1.031, *b*=1.044).

The DMIST conducted by the American College of Radiology Imaging Network was designed to measure the difference in diagnostic performance between digital and screen-film mammography for the purpose of breast-cancer screening (Pisano *et al*. 2005). In this example we used data from a retrospective reader study that randomly selected participants from DMIST study sample. Each participant underwent both digital and screen-film mammography, and mammograms from the same participant were interpreted by a radiologist using seven-point malignancy scale. Figure 8 shows empirical and estimated PROC curves from three readers. To make this example parsimonious and also to show clear trade-off between the PPV and NPV, in the following we select data from reader 3 to present an analysis that compares the predictive accuracy of digital and film mammography.

**Figure 9:**
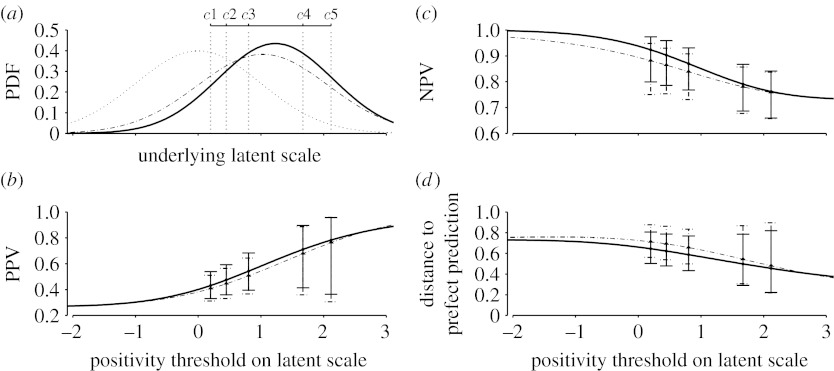
Pseudo-likelihood analysis of mammography data. (*a*) The estimated latent distributions of the diseased and non-diseased subjects under the binormal assumption with estimated operating thresholds *c*1, …, *c*5, (*b*) the estimated positive predictive value, (*c*) the estimated negative predictive value and (*d*) the estimated distance to the perfect prediction with 95% CIs. (*a*) Dotted line, non-diseased; solid line, diseased film; dot-dashed line, diseased digital. (*b–d*) Solid line, film; dot-dashed line, digital.

**Figure 10:**
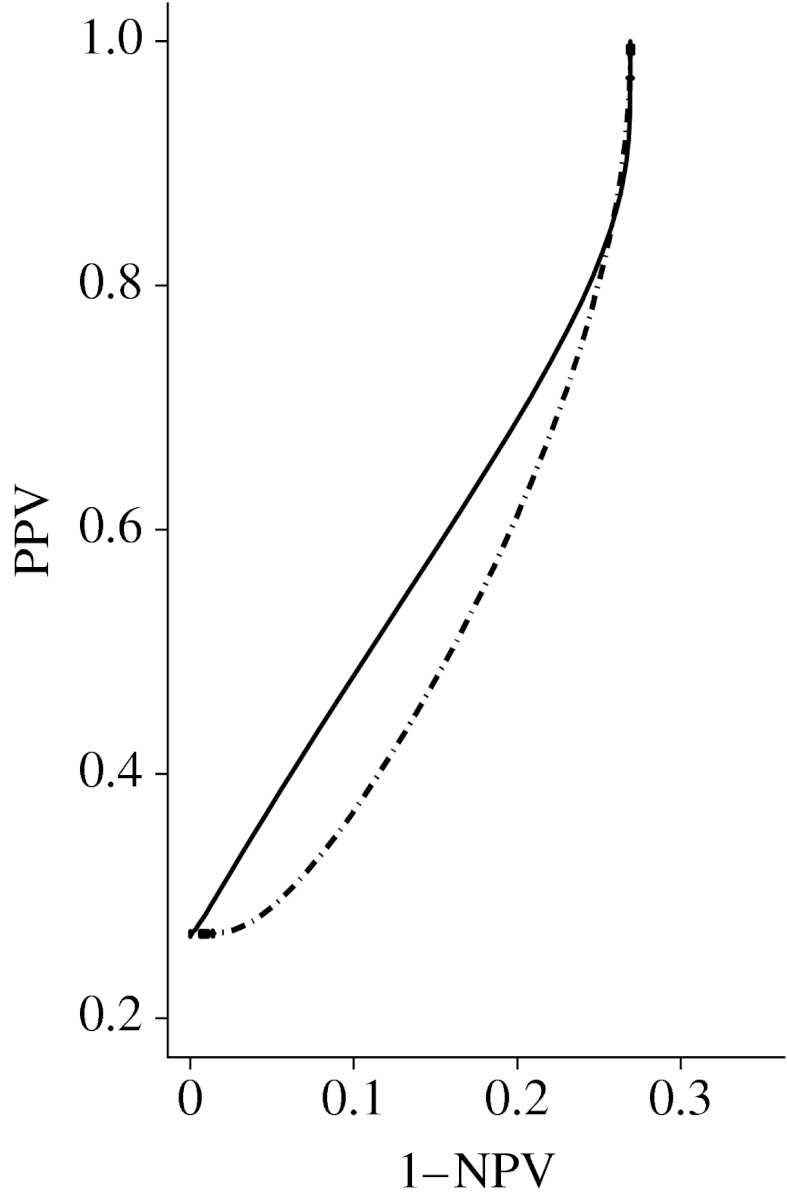
The estimated partial PROC curves of film (solid line) and digital mammography (dot-dashed line).

Among the 94 participants in the dataset, only one participant was rated as ‘7=definitely malignant’. Therefore, the seventh and sixth categories were combined for analysis purpose. The pseudo-likelihood function constructed in §4*b* was used to adjust for correlations induced by data replication and the paired study design. The delta method in the logit scale was used to calculate 95% CIs. Figure 9 presents the results of pseudo-likelihood estimation. The film mammography showed uniformly higher estimates of predictive performance than the digital mammography over all positivity thresholds. However, the difference was not statistically significant as the CIs overlapped. Figure 10 also presents the partial PROC curves without the vertical and horizontal segments that correspond to extreme positivity thresholds (beyond 15.03 and smaller than −23.71 on the latent scale). Both estimated curve segments have NPV ranging from 0.73, which is one minus the prevalence, to 1. Over this range, the average positive predictive value was 0.613 with 95% CI (0.411, 0.853) for film mammography and 0.598 with 95% CI (0.411, 0.853) for digital mammography. Lastly, the area under the loss function *R* over the range of ±2 s.d. using *α*=1 was 0.587 with 95% CI (0.471, 0.718) for film mammography and 0.632 with 95% CI (0.516, 0.762) for digital mammography. Overall, we conclude that there was no statistically significant difference in predictive accuracy between the digital and screen-film mammography.

## 7. Discussion

The unique aspect of the PROC curve is that the curve shows how PPV and NPV vary jointly as a function of the positivity threshold. Thus, the PROC curve shows all achievable pairs of PPV and NPV. In this sense, the PROC curve can be interpreted similarly to the ROC curve. The area under a monotone segment of the PROC curve can be interpreted as the average positive predictive value over a range of NPV. However, an analogue to the probabilistic interpretation of the area under the ROC curve does not seem to exist in the PROC context. In addition, in contrast to the simple trade-off between sensitivity and specificity, as captured by the ROC curve, the dependence of PPV and NPV individually and jointly on the positivity threshold does not follow simple patterns. A detailed characterization of the monotonicity of the PROC curve and its segments shows that the patterns are complex even for the binormal model. The complexity of the PROC curve reflects the complexity of the dependence between the PPV and NPV of a test. When both quantities are of interest, a separate analysis of each carries a notable element of risk for reaching misleading conclusions.

While in many situations, scientific inferences and clinical decision-making may not need to make use of the full PROC curve, such as the case when clinical interests lie in estimating the positive predictive value of a test given a certain range of NPV, or vice versa, the non-monotonicity of the full curve becomes less a concern and the monotonicity of a curve segment of interest is indeed more relevant. In situations when the full curve contains segments that correspond to positivity thresholds that are very unlikely to occur in practice, such as the visually vertical or horizontal segments in almost every PROC curve, the analyst can consider only segments of the curve instead of the full curve. Generally speaking, a PROC curve segment corresponding to positivity threshold of clinical relevance should be considered as opposed to the use of the full curve, and the properties of segments of interest can be identified through proposition 3.2. These results, however, are valid only for the binormal model. Similar investigations can be undertaken under other distributional assumptions. Non-parametric models can also be considered and may lead to more robust conclusions, as long as it is possible to verify the conditions of hazard ordering.

This paper emphasizes the importance of including the positivity threshold in an analysis of predictive values. The summary measure *r*(*c*), which retains threshold information, can be used to study the influence of the selection of a particular threshold on the predictive performance of a diagnostic test and to incorporate the threshold effect into test comparisons. The optimal operating point can also be obtained on the basis of this measure *r*(*c*). In contrast to the ROC curve that typically shows a trade-off between sensitivity and specificity, we note that in situations when *b*≠1 the PROC curve has one monotone segment of $c\in [c_{\text{PPV}}^{*},c_{\text{NPV}}^{*}]$, on which both the PPV and NPV can either increase (when *b*>1) or decrease (when *b*<1). This makes the selection of an optimal threshold on this interval a trivial task, as either the left or the right endpoint of this interval dominates the rest. However, clinical interest may not lie in this segment, as most of the observed points usually occur within the range of $c\in (-\infty ,c_{\text{PPV}}^{*})$ or of $c\in (c_{\text{NPV}}^{*},\infty )$, depending on the value of parameter *b*.

We propose summary measures such as the average loss *R* and the area under the full curve or a curve segment to represent the overall predictive performance. The average positive predictive value over a specific range of negative predictive value, or vice versa, is also useful summary measures. Regardless of which measure is chosen for the analysis, however, an understanding of the shape of the PROC curve, based on which the inference would be drawn, is still essential.

The dependence of predictive values on disease prevalence is another concern associated with the use of predictive values in the evaluation of diagnostic tests. This issue is particularly problematic when tests are to be compared on the basis of data that did not arise from a study using a paired design, even if they were prospective studies. For example, this would be ordinarily the case if data for each of the tests come from separate studies. One approach to addressing this challenge is the transformation of curves described in §5*c*. Such a transformation makes it possible to compare PROC curves at any given prevalence of interest, and therefore may be very useful in situations such as disease screening and translation of test results from one population to another. It may also be possible to incorporate covariates related to the prevalence, for example, as in the model for ordinal data presented in §4. However, our experience with the use of such modelling in empirical investigations is so far limited to relatively simple settings with binary covariates.

In conclusion, we note that although the patterns of covariation of PPV and NPV are complex, the resulting difficulty does not imply that the dependence on threshold is not important in practice. Since the clinical use of a diagnostic test is primarily based on the predictive value of the test, a thorough assessment of predictive values and their properties can provide important information.

## Supplementary Material

Web-based supplementary materials
