# Supplementary material for: The predictive receiver operating characteristic curve for the joint assessment of the positive and negative predictive values
Source: Philos Trans A Math Phys Eng Sci. 2008 Apr 11;366(1874):2313–33. doi: 10.1098/rsta.2008.0043 (PMC3227148; doi:10.1098/rsta.2008.0043)
Supplement: Web-based supplementary materials [file rsta20080043s01.pdf]

Web-based Supplementary Materials

The Predictive Receiver Operating Characteristic Curve for the Joint Assessment of the Positive and Negative Predictive Value

SHANG-YING SHIU

Institute of Statistical Science, Academia Sinica, Taipei 115, Taiwan

CONSTANTINE GATSONIS

Center for Statistical Sciences, Brown University,

Providence, RI 02912, U.S.A.

Appendix A

An interpretation of the hazard rate order

In this appendix we give an interpretation of the hazard rate order and provide an example of when the condition fails. Similar argument can apply to the reversed hazard rate order.

Definition 1. *(The Hazard Rate Order) Let X and Y be two random variables with absolutely continuous distribution functions F and G, respectively, such that*

$$\frac{f(t)}{1-F(t)} \leq \frac{g(t)}{1-G(t)}, \quad \forall t > -\infty.$$

Then X is said to be smaller than Y in the hazard rate order.

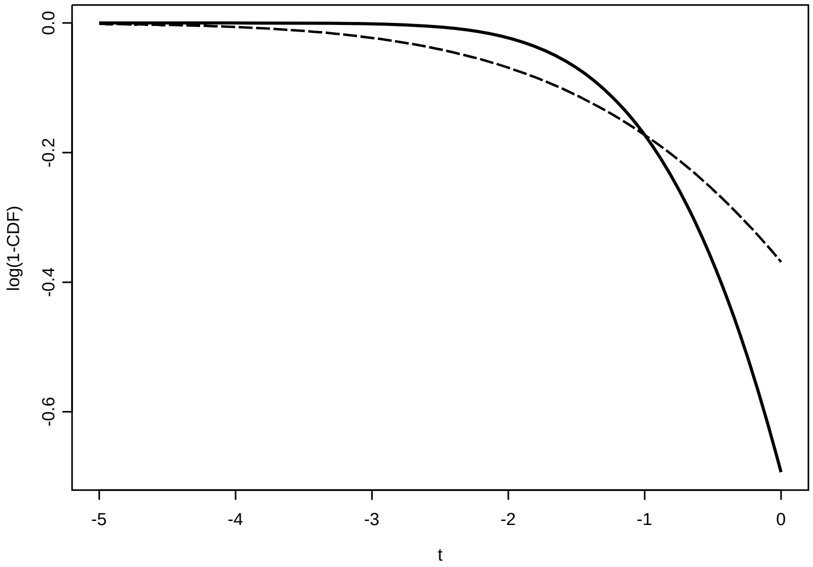

Figure 1. A violation of the hazard rate order: the binormal assumption with $a = 1$ and $b = 2$.

The hazard rate order is defined on the ratio of the PDF and the survival function. This ratio $f(t)/(1 - F(t))$ is the first derivative of $log(1 - F(t))$. Therefore, the hazard rate order implies that the slope of the log of one survival function is uniformly smaller than that of the other.

If one function does not have a uniformly smaller slope than the other, and if the two functions have the same starting points (which are zero here), then for the function initially having a lower speed therefore falling behind at the beginning may pass over the other function because of a higher speed later. In other words, the two functions are likely to cross at some point. Figure 1 demonstrates the failure of the hazard rate order. The solid curve represents the log of the survival function from N(0,1), and the dashed curve

is from N(1,2). Note that the two curves cross at some point around minus one.

To summarize, one interpretation of the hazard rate order is that the log of the survival function of one random variable declines uniformly faster than that of the other. A common example that the hazard rate order fails, is that the log of the survival functions of two random variables cross. However, the non-crossing of the log of the two survival functions does not imply that the hazard rate order is satisfied.

Appendix B

Proofs of Proposition 3.1 and Proposition 3.2

In this appendix, we will prove Proposition 3.1 and Proposition 3.2. For convenience of notation, let

$$h^+_{a,b}(t) = \frac{1-\Phi(\frac{t-a}{b})}{\phi(\frac{t-a}{b})/b} \quad \text{and} \quad h^-_{a,b}(t) = \frac{\Phi(\frac{t-a}{b})}{\phi(\frac{t-a}{b})/b}.$$

Then we have

$$\begin{aligned} \frac{d}{dt}PPV(t) \geq 0 \quad &\Longleftrightarrow \quad h^+_{a,b}(t) \geq h^+_{0,1}(t), \\ \frac{d}{dt}NPV(t) \geq 0 \quad &\Longleftrightarrow \quad h^-_{a,b}(t) \geq h^-_{0,1}(t). \end{aligned}$$

We begin with studying the geometric properties of $h^+_{a,b}$ and $h^-_{a,b}$. The following lemma describes the properties, and will be used to prove Proposition 3.1 and Proposition 3.2.

Lemma 1. $\forall a, b > 0$,

(i) $h^{+}_{a,b}$ *is strictly decreasing and convex on* $t \in (-\infty, \infty)$.

(ii) $h^{-}_{a,b}$ *is strictly increasing and convex on* $t \in (-\infty, \infty)$.

Proof. In the following, we only prove statement (i). Statement (ii) can be obtained by similar arguments.

It is equivalent to prove that $h^{+}_{0,1}$ is strictly decreasing and convex. By taking the first derivative of $h^{+}_{0,1}$, we have

$$\frac{d}{dt}h^{+}_{0,1}(t) = \frac{g(t)}{\phi(t)},$$

where $g(t) = t[1 - \Phi(t)] - \phi(t)$.

Since

$$\frac{d}{dt}g(t) = 1 - \Phi(t) > 0,$$

and since

$$\lim_{t\to\infty} g(t) = \lim_{t\to\infty} -\phi(t) + \frac{t}{\frac{1}{1-\Phi(t)}} = 0,$$

we obtain that $g(t) < 0\ \forall t \in (-\infty, \infty)$. Therefore $h^{+}_{0,1}$ is strictly decreasing. By taking the second derivative of $h^{+}_{0,1}$, we have

$$\frac{d^2}{dt^2}h^{+}_{0,1}(t) = \frac{f(t)}{\phi(t)},$$

where $f(t) = 1 - \Phi(t) + g(t)t$.

Since

$$\frac{d}{dt}f(t) = 2g(t) < 0,$$

and since

$$\lim_{t\to\infty} f(t) = \lim_{t\to\infty} 1 - \Phi(t) + \frac{t}{\frac{1}{g(t)}} = 0,$$

we obtain that $f(t) > 0\ \forall t \in (-\infty, \infty)$. Therefore $h^+_{0,1}$ is convex. $\square$

Proof of Proposition 3.1. Since $h^+_{a,b}$ is strictly decreasing, and since $h^-_{a,b}$ is strictly increasing, we have

$$\begin{aligned} h^+_{a,1}(t) &= h^+_{0,1}(t-a) > h^+_{0,1}(t) \quad \forall t, \\ h^-_{a,1}(t) &= h^-_{0,1}(t-a) < h^-_{0,1}(t) \quad \forall t. \end{aligned}$$

These two inequalities complete the proof. $\square$

Proof of Proposition 3.2. In the following, we will only prove Proposition 3.2 for $b > 1$. The statements for $b < 1$ can be obtained using similar arguments.

(a) Since $b > 1$, we have

$$1 - \Phi(\frac{t-a}{b}) < 1 - \Phi(t) \quad \forall t < c_1^*,$$

where $c_1^* = a/(1-b)$. Moreover, since

$$\frac{\phi(\frac{t-a}{b})/b}{\phi(t)} = \frac{1}{b} e^{-\frac{1}{2}(\frac{1}{b^2}-1)t^2 + \frac{a}{b}t - \frac{1}{2}(\frac{a}{b})^2} \to \infty \quad \text{as} \quad t \to -\infty,$$

that is,

$$\exists c_2^* < 0 \quad \text{such that} \quad \frac{\phi(t)}{\phi(\frac{t-a}{b})/b} < 1 \quad \forall t < c_2^*.$$

Therefore, we have $h^+_{a,b}(t) < h^+_{0,1}(t)\ \forall t < min(c_1^*, c_2^*)$.

(b) Since $h^+_{a,b}(t)$ is decreasing and $b > 1$, we have

$$h^+_{a,b}(t) = bh^+_{0,1}(\frac{t-a}{b}) \geq bh^+_{0,1}(t) > h^+_{0,1}(t) \quad \forall t \geq \frac{a}{1-b}.$$

(c) Since $h^{+'}_{a,b}$ is increasing, and since

$$h^{+'}_{a,b}(t) = h^{+'}_{0,1}(\frac{t-a}{b}),$$

we have

$$\begin{aligned} h^{+'}_{a,b}(t) &> h^{+'}_{0,1}(t), \quad \forall t < \frac{a}{1-b}, \\ h^{+'}_{a,b}(t) &= h^{+'}_{0,1}(t), \quad t = \frac{a}{1-b}, \\ h^{+'}_{a,b}(t) &< h^{+'}_{0,1}(t), \quad \forall t > \frac{a}{1-b}. \end{aligned}$$

Combine results from (a), (b) and (c), we conclude that there exists a unique $c^*_{PPV} \in (min(c^*_1, c^*_2), a/(1-b))$ such that

$$\begin{aligned} h^{+}_{a,b}(t) &< h^{+}_{0,1}(t) \quad \forall t < c^*_{PPV}, \\ h^{+}_{a,b}(t) &> h^{+}_{0,1}(t) \quad \forall t > c^*_{PPV}. \end{aligned}$$

Therefore, the positive predictive value is strictly decreasing on $t \in (-\infty, c^*_{PPV})$ and is strictly increasing on $t \in (c^*_{PPV}, \infty)$.

Similarly, we can prove that the negative predictive value is strictly increasing on $t \in (-\infty, c^*_{NPV})$ and is strictly decreasing on $t \in (c^*_{NPV}, \infty)$, where $c^*_{NPV} > a/(1-b)$. Therefore, we also conclude that $c^*_{PPV} \leq c^*_{NPV}$. □

Appendix C

Existence of pseudo-likelihood estimates and the variance-covariance matrix

(i) The existence of pseudo-likelihood estimates depends on the identifiability of the model. For the predictive model presented in Section 4, the identifiability is guaranteed whenever the number of ordinal categories $K \geq 3$.

(ii) Examine if the matrix

$$J = -\sum_{k=1}^{K} \delta_k E_\Psi \left(\frac{\partial^2 lnf_k(D|I_k;\Psi)}{\partial\psi_u \partial\psi_l} \right)$$

is positive definite, where $\Psi = \{\theta_1, ..., \theta_{k-1}, a, b, r\}$,

$$lnf_k(D|I_k;\Psi) = -Dln(1+h_k) + (1-D)ln(1 - \frac{1}{1+h_k}),$$

and $h_k = r(I_k - \Phi(\theta_k))/(I_k - \Phi((\theta_k - a)/b)$.

Since

$$\frac{\partial lnf_k(D|I_k;\Psi)}{\partial\psi_u} = \frac{\partial lnf_k(D|I_k;\Psi)}{\partial h_k}\frac{\partial h_k}{\partial\psi_u} = \left[-\frac{1}{1+h_k} + (1-D)\frac{1}{h_k} \right] \frac{\partial h_k}{\partial\psi_u},$$

the expected second derivative is

$$\begin{aligned} -E_\Psi \left(\frac{\partial^2 lnf_k(D|I_k;\Psi)}{\partial\psi_u \partial\psi_l} \right) &= E_\Psi \left(\frac{\partial lnf_k(D|I_k;\Psi)}{\partial\psi_u} \frac{\partial lnf_k(D|I_k;\Psi)}{\partial\psi_l} \right) \\ &= \frac{\partial h_k}{\partial\psi_u}\frac{\partial h_k}{\partial\psi_l} E_\Psi \left(-\frac{1}{1+h_k} + (1-D)\frac{1}{h_k} \right)^2, \end{aligned}$$

which shows that the matrix J is of the form $\sum V_k V_k^T c_k$, where V_k is the vector of $\partial h_k/\partial\Psi$ and c_k is a constant larger than zero. Hence, it is proved that J is positive definite.
